# Continuous Multi-DoF Wrist Kinematics Estimation Based on a Human–Machine Interface With Electrical-Impedance-Tomography

**DOI:** 10.3389/fnbot.2021.734525

**Published:** 2021-09-30

**Authors:** Enhao Zheng, Jingzhi Zhang, Qining Wang, Hong Qiao

**Affiliations:** ^1^The State Key Laboratory for Management and Control of Complex Systems, Institute of Automation, Chinese Academy of Sciences, Beijing, China; ^2^School of General Engineering, Beihang University, Beijing, China; ^3^Department of Advanced Manufacturing and Robotics, College of Engineering, Peking University, Beijing, China

**Keywords:** wrist angle estimation, electrical-impedance-tomography, multi-DoF, Lasso, human-machine interface

## Abstract

This study proposed a multiple degree-of-freedom (DoF) continuous wrist angle estimation approach based on an electrical impedance tomography (EIT) interface. The interface can inspect the spatial information of deep muscles with a soft elastic fabric sensing band, extending the measurement scope of the existing muscle-signal-based sensors. The designed estimation algorithm first extracted the mutual correlation of the EIT regions with a kernel function, and second used a regularization procedure to select the optimal coefficients. We evaluated the method with different features and regression models on 12 healthy subjects when they performed six basic wrist joint motions. The average root-mean-square error of the 3-DoF estimation task was 7.62°, and the average *R*^2^ was 0.92. The results are comparable to state-of-the-art with sEMG signals in multi-DoF tasks. Future endeavors will be paid in this new direction to get more promising results.

## 1. Introduction

Human–machine interface with muscle signals for wrist kinematics recognition/decoding/estimation is a key part in wearable exoskeletons, robotic prosthesis, and human–robot collaborations (Farina et al., 2014; Accogli et al., 2017; Peternel et al., 2017; Fani et al., 2018; Kapelner et al., 2019; Hussain et al., 2020). It is an effective way to bridge the gap between the human sensorimotor system and robotic devices. Compared with the mechanical signals, such as inertial measurement units, which respond to the human motions, the muscle signals convey their sources. They can be extracted in advance of the actual motions (Scott, 2004). The recognized wrist kinematic information can serve as the control inputs to the robotic controllers to provide motion initiation/termination (Hussain et al., 2020), targeted gestures (Farina et al., 2014; Kapelner et al., 2019), and continuous motion parameters (angles and velocities) (Peternel et al., 2017; Fani et al., 2018).

Due to the technological development, the surface electromyography (sEMG) is the most widely used muscle signal in wearable robotics (Novak and Riener, 2015; Rodríguez-Tapia et al., 2020). The sEMG sensors can measure muscular (superficial muscles) electric activities from the surface of the skin. The wrist motion information can be extracted by measuring the sEMG signals from the forearm and the subsequent processing algorithms. The studies on sEMG-based wrist kinematics estimation/recognition/decoding vary according to the tasks and motivations (robotic platforms) (Rodríguez-Tapia et al., 2020). There are continuous wrist angle estimation and target-oriented posture recognition (mostly discrete patterns), which determines the subsequent algorithms and experimental validations. For research motivations, wrist kinematics estimation can be applied in robotic prostheses, exoskeletons, and manipulators. The participants include healthy persons, stroke survivors, and upper-limb amputees. The evaluation metrics are also different according to the tasks. For continuous wrist kinematics estimation (the target of our study), the researchers of sEMG-based studies used the coefficient of determination (*R*^2^) and root mean square errors (RMSEs) to evaluate the estimation performances (Bi et al., 2019). For continuous wrist kinematic estimation, the number of degrees of freedom (DoFs) is a major factor to influence the estimation performances (Muceli and Farina, 2012; Liu et al., 2017; Bakshi et al., 2018; Gao et al., 2018; Kapelner et al., 2019; Shahzad et al., 2019; Yang et al., 2019; Ameri, 2020; Bao et al., 2020, 2021; Zhao et al., 2020). Simultaneous multi-DoF estimation is still a challenging task for muscle-signal-based studies. Among state-of-the-art of sEMG-based studies, the average estimation accuracies (represented by *R*^2^) of 1-DoF wrist angle estimation were higher than that of multi-DoFs. One reason for the difficulty is that human forearm muscles are coupled in controlling the multi-DoF wrist motions. To increase the performances in multi-DoF tasks, the researchers designed algorithms to extract more myoelectric features or developed high-density sEMG systems to extract the features of motor unit potentials. For instances, the studies of Muceli and Farina (2012) and Shahzad et al. (2019) sampled forearm muscle signals with high-density (HD)-EMG system and achieved simultaneous 3-DoF (flexion/extension, ulna/radius deviation, and pronation/supination) angle estimation. Due to the numerous signal channels and much neural information, the researchers designed estimation algorithms by an artificial neural network (ANN) with principal component analysis (PCA) or motor neuron discharge timing-related neural features. Due to the complexity of the task, the average *R*^2^ values ranged from 0.77 to 0.88 with different parameters across 6 healthy subjects.

On the other side, the sEMG sensors can only measure the information of superficial muscles (Vigotsky et al., 2018). The deep muscle contractions also contain abundant human motion information. Some researchers proposed using different signal sources to extract more motion features. Some researchers in this field use ultrasound (US) imaging techniques to measure muscle anatomy structures (Shi et al., 2009; Huang and Ono, 2016; Kato et al., 2018; Yang et al., 2020b). The US imaging technique can reconstruct the muscle morphological information of the scanning region (including the deep muscles). According to the sensing principles of US devices, the spatial resolution of the US images is high. The muscle architecture constructed by the US technique is regarded as the ground truth for assessment in physical therapy and rehabilitation. Some researchers are conducting US-based studies on forearm motion estimation/recognition tasks, such as finger angle/force estimation and gesture classification. On the other side, the US device is burdensome in the probe and the signal processing system. The sensing front-end of the US device is made up of rigid material (mostly piezoceramics), which is not suitable for wearing. Some researchers developed the wearable US front-ends for human–machine interfaces to increase the compactness of the technique for wearable uses, but the imaging property is simplified (Yang et al., 2020b). Another technique to inspect deep muscle information in a non-invasive way is the electrical impedance tomography (EIT). The EIT technique stimulates and measures from the surface of the medium with a predefined order to reconstruct the conductivity distribution inside the medium. The technique is free from ionizing radiation compared with the computational tomography (CT) technique. Previous EIT-based studies were focused on physical condition monitoring, including respiration monitoring (lung) and tumor detection (Frerichs et al., 2017). With the development of integrated circuits, the researchers began using the EIT technique for human–machine interfaces. The tasks were focused on discrete gesture recognition, and the studies produced comparable results to that of sEMG-based approaches (Zhang et al., 2016; Wu et al., 2018; Ma et al., 2020).

We recently proposed an EIT-based interface for continuous forearm motion estimation, including grasp force estimation and wrist flexion/extension angle estimation. Compared with the previous works on EIT-based forearm motion recognition, our recent works addressed the issues of continuous motion estimation (Zheng et al., 2019, 2020, 2021). In the studies of Zheng et al. (2019, 2020), we designed an EIT-based interface for continuous grasp force estimation and human–robot co-manipulation. The average *R*^2^ value was 0.9 in the off-line task, and the interface accomplished human–robot sawing with varying sawing frequency, forces, and sawing direction. In a more recent work (Zheng et al., 2021), we proposed an EIT-driven musculoskeletal model to map the forearm EIT signals to the wrist joint flexion/extension angles. Unlike the state-of-art on EIT-based human motion recognition, we built a forward musculoskeletal model from the EIT signal features to the wrist joint angles by taking advantage of the muscle spatial information. However, our previous work on EIT-based wrist kinematics estimation was a 1-DoF task (flexion/extension). In our current study, we proposed a multi-DoF wrist angle estimation approach based on the EIT interface. The interface, on the one hand, extends the measurement range of sEMG sensors for deep muscle spatial information, and, on the other hand, reduces the burdensome sensing front-ends of US probes by the soft elastic fabric band. The designed approach took advantage of the anatomical features within the EIT signals by the specifically designed features and regression models. Compared with our previous works, the approach can simultaneously output the 3-DoF wrist joint angles. We then evaluated the proposed method with experiments of multi-DoF wrist joint motions.

## 2. Measurement System

**Figure 1:**
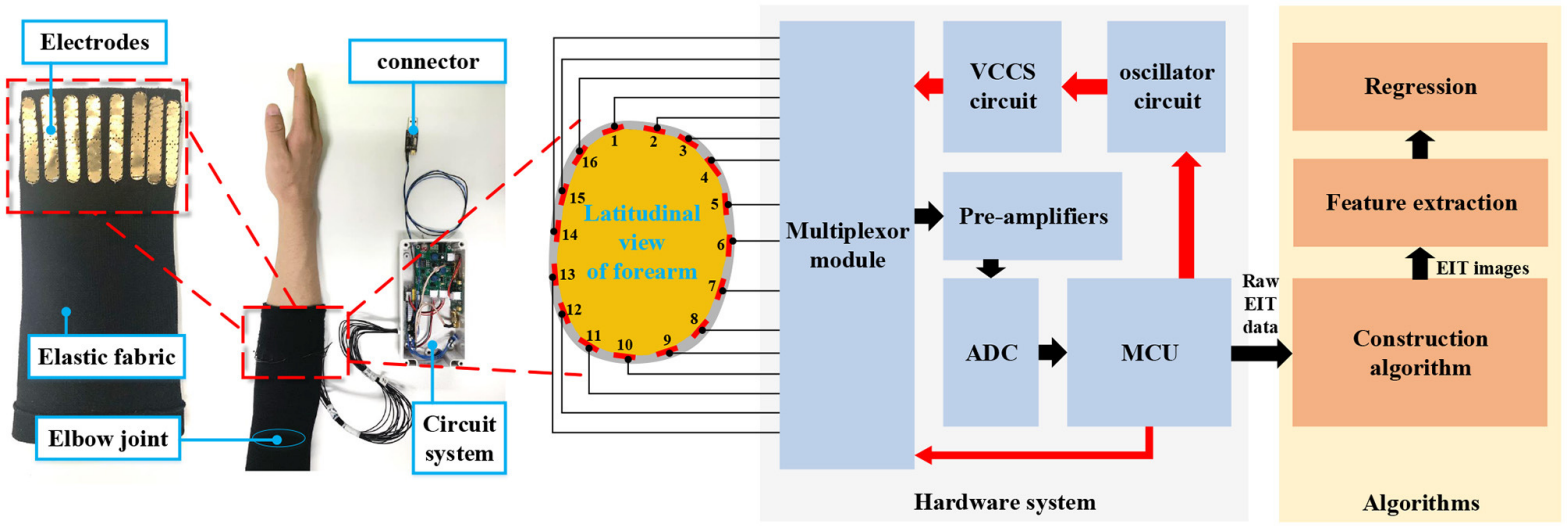
The diagram of the EIT-based wrist estimation. The measurement system comprises the hardware system and the algorithms. The red arrows denote the control signals/commands while the black arrows denote the measured signals/data. The MCU outputs the raw EIT data for subsequent algorithms. The left part of the figure shows the actual EIT system, including the sensing front-end (with inside out) and the circuit system. The structure of the sensing front-end in latitudinal view is shown in the middle part of the figure. The red bars in the gray closed band indicate the electrodes inserted on the sensing band. Each of them is denoted by a number (1–16).

The measurement system was designed with the sensing front-end, the hardware system (sensing circuit system), and the construction algorithms. The sensing front-end was an elastic fabric band with 16 electrodes fixed on the inner surface (left part of Figure 1). The electrodes were evenly distributed in the latitudinal plane of the human arm. Each electrode was a flexible printed circuit board (flex-PCB). The circuit system connects with the sensing front ends via the shielding lines. The hardware system stimulates the electrodes with a current signal, and measures the voltages on the other electrodes. We stimulated and measured adjacent electrode pairs in our system. With this stimulation/measurement method, there were 16 stimulations, and each stimulation had 13 measurements to get data for one EIT image. The stimulation current signal was s 40-kHz sinusoid signal generated by the oscillation circuit and the voltage-controlled current source (VCCS). The root-mean-square (RMS) of the current was 400 μA. The voltage signals on the other electrode pairs went through pre-amplifying and analog-to-digital (ADC) sampling. The routing of the stimulation and measurements was accomplished with the multiplexor module. The micro-control unit (MCU) in the circuit system calculated the RMS value of the measured voltages (one for each channel in a sample). The raw EIT data consisted of 208 voltages in each sample. The sampling frequency was 10 Hz.

The task of the construction algorithm was to calculate the tissue conductivity (inverse of the impedance) distribution within the scanning plane based on the voltages measured from the surface. It was an inverse problem that conformed to the Dirichlet Boundary Condition. According to Maxwell's equations with proper assumptions and approximations, the electrical property within the surface (human skin) was expressed as:


(1) $$\begin{array}{l} \nabla \cdot \sigma \nabla \phi =0, \end{array}$$

where *ϕ* is the electrical potential distribution, *σ* is the conductivity distribution, and ∇ is the Hamiltonian operator. The electrical property at the boundary was stated as *σ*∇***n*** = *J*_*n*_, where ***n*** is the unit normal vector of the boundary and *J*_*n*_ is the current density at the *n*′*s* electrode. To acquire the conductivity σ with the measured signals on the electrodes, we designed two stages calculation, i.e., the forward model construction and the image reconstruction.

To calculate the differential equations, we implemented the forward model based on the finite element method (FEM). We first built a closed shape as the boundary indicating the forearm cross-sectional view. To compensate for the shape mismatch in the model construction, we built the shape of the area by considering the forearm geometry. The shape of the boundary was more similar to the latitudinal view of the forearm compared to a simple circle. *N* triangles were used to segment the area into small nodes. The forward model based on FEM could be expressed as:


(2) $$\begin{array}{l} V=F\left(\sigma \right), \end{array}$$

where ***σ*** was a vector with *N* dimensions, and each element was the conductivity of the corresponding node in the FEM model. ***V*** was the voltage on the surface of the body. The forward model was to calculate ***V*** from the known ***σ***.

With the built forward model (Equation 2), we could obtain the conductivity distribution ***σ*** by minimizing the square errors:


(3) $$\begin{array}{l} \min\left(\left\|V_{m}-F\left(\sigma \right)\right\|\right), \end{array}$$

where ***V***_***m***_ is the measured voltages by repeated stimulation and measuring. *F*(***σ***) is the constructed forward model that mapped conductivity to the surface voltages. It was a non-linear problem to calculate ***σ***. There were several ways to linearize the calculation. In this study, we used time-different EIT (tdEIT) method, in which a baseline conductivity ***σ***_**0**_ was predefined. The relation near the baseline could be expressed as:


(4) $$\begin{array}{l} V_{m}=F\left(\sigma_{0}\right)+\frac{\partial F\left(\sigma_{0}\right)}{\partial \sigma }\left(\sigma -\sigma_{0}\right). \end{array}$$

By substituting the partial derivative with the matrix ***J***, the equation was converted to:


(5) $$\begin{array}{l} \delta \text{V}=\text{J}\delta \sigma , \end{array}$$

where δ***V*** and ***σ*** were the small variations of the voltage and conductivity at the baseline.

By the inverse of the Jacobian matrix ***J***, the conductivity distribution could be calculated. As ***J*** was not a square matrix, pseudo inverse matrix was used for calculation (Adler and Guardo, 1996). It was expressed as:


(6) $$\begin{array}{l} \delta \sigma =\left(J^{t}WJ+\lambda^{2}Q\right)J^{t}W\delta V, \end{array}$$

where ***J***^***t***^ is the transposition, and ***W*** and ***Q*** are the constructed square matrices and λ was the hyper parameter. The algorithms were implemented on the computer with MATLAB2016b. The EIDORS toolkit was used to provide the functions for solving the differential equations^1^. The forward model construction (FEM model with the predefined boundary) was implemented off-line before the measurement. The baseline conductivity **σ_0_** and the Jacobian matrix ***J*** were also calculated off-line with the initial measured voltages ***V***_**0**_. During online calculation, Equation (6) is calculated with the update of the measured voltages ***V***_***m***_ in each 100 ms.

## 3. Methods

### 3.1. Experimental Protocol

**Figure 2:**
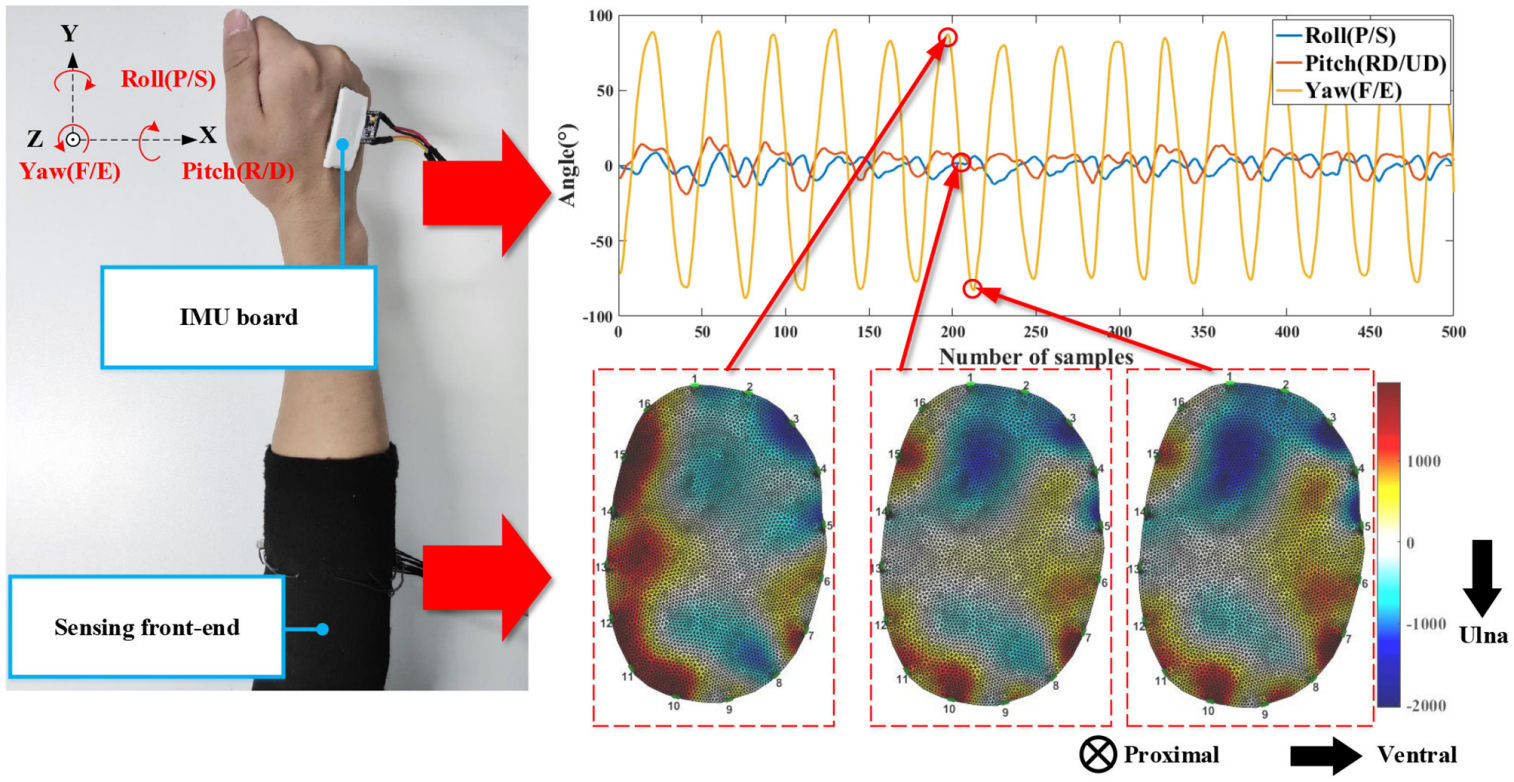
The experimental setups **(left)** and the typical signals of the measurement system **(right)**. The IMU board was fixed on the back of the palm. The IMU board outputs 3-axis angles (pitch, roll, yaw). F/E represents flexion/extension, RD/UD represents radius/ulna deviation, and P/S represents pronation/supination. The three EIT images (from the **left** to **right**) correspond to the maximum extension angle, the neutral state, and the maximum flexion angle. The EIT images show the cross-sectional conductivity distribution of the measured forearm. The anatomical direction is shown with the arrows.

Twelve male subjects participated in the experiment. All of them provided written and informed consent. The experiment was approved by the Ethical Review Board of Institute of Automation, Chinese Academy of Sciences (No. IA-202008). They had an average age of 24.5 ± 2.39 years, an average height of 173.2 ± 4.90 cm, and an average weight of 70.2 ± 5.89 kg. All data were recorded with their right forearm. We investigated the geometry parameters of the measured forearm. The average perimeter of their measured forearm (maximum part) was 25.7 ± 1.27 cm. The average length of the forearm was 24.0 ± 1.92 cm. The subjects wore the sensing front-end and an IMU board on the measured forearm (see left part of Figure 2). The IMU board adhered to the back of the palm via double-sided paste. During the experiment, three-DoF wrist motions were measured, i.e., the flexion/extension, pronation/supination, and radius/ulnar deviation. The subjects started from the neutral position with the palm vertical to the ground and performed the motion of each DoF repeatedly at their own pace. They also performed the motion of each DoF by reaching the maximum extent. We measured two sessions, and in each session, the subjects performed 20 cycles for each DoF (60 cycles in total). They rested for 10–20 min between the sessions. The EIT board output the tilt angles (pitch, roll, yaw). The relation between the tilt angles and the wrist motions is shown in the right part of Figure 2. The sampling rate of EIT system was 10 Hz, and the sampling rate of the IMU sensor was 100 Hz. During the experiment, the IMU sensor was down sampled 10 Hz to synchronize with the EIT system.

### 3.2. Feature Extraction

In our study, the feature extraction comprises the EIT image segmentation, feature calculation, and regularization. In our study, one EIT image is a closed shape comprising 5,868 FEM nodes (right bottom of Figure 2). The nodes' values convey both temporal and spatial information of the anatomical cross-sectional plane. We first down-sampled the EIT image of each frame ***z*** ∈ ℝ^*N*×1^ into *M* small square regions with equal sizes. In our study, N = 5,868 and *M* = 382. We calculated the average values in each region as the EIT features for the subsequent procedure, which was expressed as:


(7) $$\begin{array}{l} S\left(i\right)=\frac{1}{K\left(i\right)}\underset{\left(x,y\right)\in \Omega \left(i\right)}{\sum }z\left(x,y\right), \end{array}$$

where ***z***(*x, y*) is the re-constructed value of the FEM node in Cartesian coordinate system, Ω(*i*) is the *i*_*th*_, (*i* = 1, 2, 3, …, *M*) segmented region, and *K*(*i*) is the total number of the FEM nodes in Ω(*i*). ***S***(*i*) was the feature of the *i*_*th*_ region.

**Figure 3:**
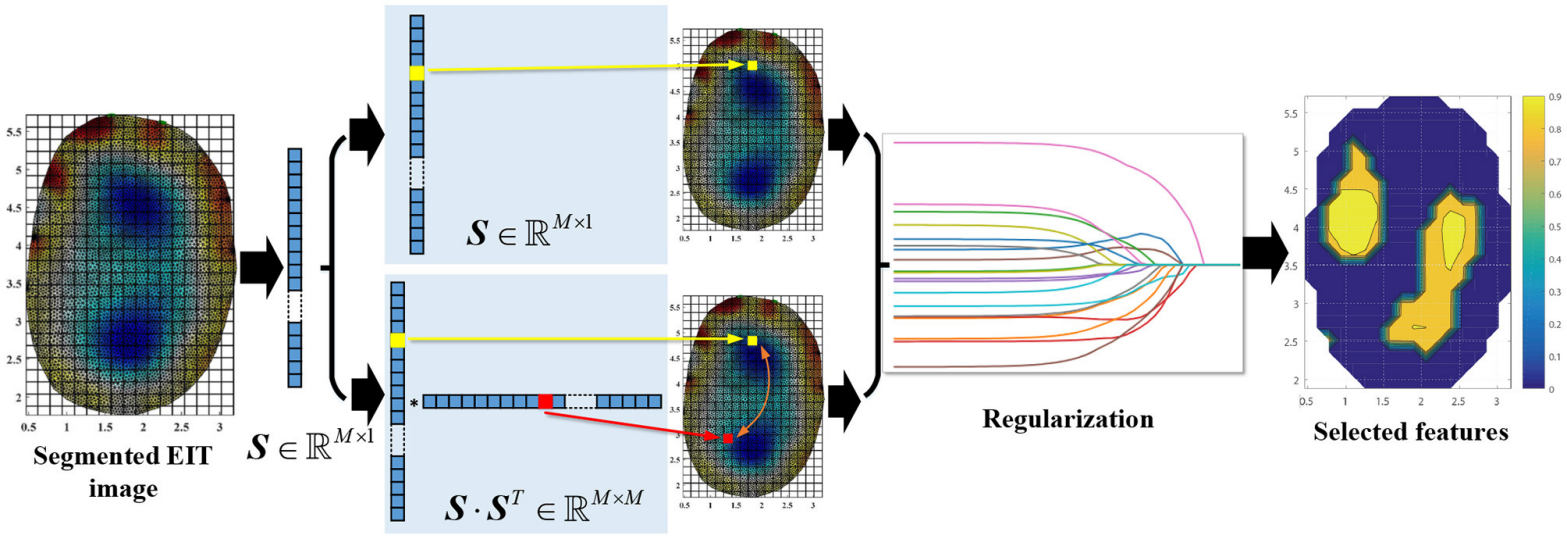
The procedures of feature extraction. ***S*** was the segmented feature and *M* = 382. The EIT images show the cross-sectional conductivity distribution of the measured forearm. The anatomical direction is the same as shown in Figure 2. The right plot denotes the selected EIT features with different estimation results (coefficient of determination, which will be described below in details). Warm colors represent higher values and cold colors represent smaller ones.

The feature calculation worked together with the regularization procedure. Our previous works (Zheng et al., 2019, 2020) used the exhaustion method to select the optimal regions in grasp force estimation and 1-DoF angle estimation (flexion/extension). In the method, ***S***(*i*) (*i* = 1, 2, …, 382) went through the regression models (sigmoid function or musculoskeletal-model-based model) to obtain the estimation results. The region with the best results served as the optimal region for estimation on the testing data set. The selected optimal region produced accurate results in 1-DoF tasks (wrist angle and grasp force). However, the multi-DoF wrist motions were coordinated by multiple forearm muscles. We used a regularization algorithm to select the optimal regions to extract more spatial information from the EIT signals. The hyperparameters determined the number of regions in the regularization algorithm (described in detail below). We designed two methods for feature calculation (Figure 3). In the first method, the features ***S*** ∈ ℝ^*M*×1^ directly went through the regularization. In the second method, we took advantage of the mutual information of different regions. The feature $\overset{-}{S}$ was expressed as:


(8) $$\begin{array}{l} \overset{-}{S}=vec\left(triu\left(S\cdot S^{T}\right)\right), \end{array}$$

where ***S*** ∈ ℝ^*M*×1^ is the segmented EIT feature as mentioned above, and ***S***^*T*^ is the transpose of the feature vector. *triu*(·) represented the upper-triangular matrix. *vec*(·) represented the vectorization of the matrix. In the following analysis, the upper-triangular matrix was expressed as: ***S***^¬^ = *triu*(***S***·***S***^*T*^). The augmented feature $\overset{-}{S}$ was a vector with $\frac{M\left(M+1\right)}{2}$ elements.

### 3.3. Regression Methods

#### 3.3.1. Lasso With Generalized Linear Model

We used least absolute shrinkage and selection operator (Lasso) for angle estimation and regularization. Lasso predicts the wrist angle with maximum-likelihood fitted parameters and proper penalization. The fitting target of Lasso was to minimize the loss function expressed as:


(9) $$\begin{array}{l} \underset{\beta ,\beta_{0}}{\min}\frac{1}{2N}[\sum_{i=1}^{N}d(x_{i},y_{i})+\lambda \sum_{j=0}^{N}\left|\beta_{j}\right|], \end{array}$$

where *N* is the number of observations in the training set, *M* is the dimension of the observation, β_*j*_ is the *j*_*th*_ weight of the observation, $y_{i}\in {\mathbb{R}}^{3\times 1}$ is the *i*_*th*_ observation (wrist angles) of the training set, and λ is the parameter to adjust the weight of the penalty term $\overset{M}{\underset{j=0}{\sum }}|\beta_{j}|$. ***x***_*i*_ is the *i*_*th*_ observation of the features. The input data ***x***_*i*_ is selected as ***S*** or augmented feature $\overset{-}{S}$ according to the feature calculation methods mentioned above. The first term $D\left(Ŷ,Y\right)=\overset{N}{\underset{i=1}{\sum }}d\left({ŷ}_{i},y_{i}\right)$ was the deviance between the actual observations ***y***_*i*_ and estimated ones ***ŷ***_*i*_. The deviance is usually expressed with log-likelihood form. In our study, we used generalized linear model with normal distribution assumption. The deviance was expressed as:


(10) $$\begin{array}{l} D(\hat{Y},Y)=\sum_{i=1}^{N}(\beta^{\prime }x_{i}+{\beta^{\prime }}_{0}-y_{i}{)}^{2}, \end{array}$$

where ***β′*** is the weight and $\beta_{0}^{\prime }$ is the intercept. The prediction function of Lasso is $ŷ=\beta^{\prime }x+\beta_{0}^{\prime }$, where ***ŷ*** is the estimated wrist angle, and ***x*** is the input data for prediction. In Equation (9), the deviance term is building the relationships between wrist angle and the EIT features. The L1-norm term is actually selecting the optimal regions from the EIT signals. The weight matrix ***β′*** was a sparse matrix, and the non-zero elements actually worked in wrist angle estimation. In our study, to reduce the computational burden, we trained three models, one for each DoF (yaw, pitch, and roll). The prediction function can be expressed as: $\hat{y_{k}}=\beta_{k}^{\prime }x+\beta_{0k}^{\prime }$, *k* = 1, 2, 3, where $\hat{y_{k}}$ is the estimated angle of the *k*_*th*_ DoF, $\beta_{k}^{\prime }$ is the *k*_*th*_ weight vector, and $\beta_{0k}^{\prime }$ is the corresponding intercept.

#### 3.3.2. SVR

The prediction function of the kernel-based support vector regressor (SVR) is *ŷ* = ***ωϕ***(***x***) + *b*, where **ϕ**(***x***) is the input data with kernel function, *ŷ* is the estimated wrist angle, ***ω*** is the weight, and *b* is the intercept. The input data ***x*** was the feature vector, which was selected as ***S*** or augmented feature $\overset{-}{S}$ according to the feature calculation methods mentioned above. The kernel function was the radial basis function, which was expressed as: $\kappa \left(x_{n},x\right)=exp\left(-\sigma {\left\|x_{n}-x\right\|}^{2}\right)$. The SVM for regression is to solve the optimization problem:


(11) $$\begin{array}{l} \underset{\omega ,b}{\min}\frac{{\left\|\omega \right\|}^{2}}{2}+C\overset{N}{\underset{i=1}{\sum }}\left(\xi_{i}+\xi_{i}^{*}\right),\\ s.t.:\\ \;\xi_{i}\geq 0,\\ \;\xi_{i}^{*}\geq 0,\\ \;y_{i}-\left(\omega \phi \left(x_{i}\right)+b\right)-\xi_{i}\leq \epsilon ,\\ \;\left(\omega \phi \left(x_{i}\right)+b\right)-y_{i}-\xi_{i}^{*}\leq \epsilon , \end{array}$$

where ϵ is the deviation between the estimated angle and the actual one, ξ_*i*_ and $\xi_{i}^{*}=E_{\epsilon }\left({ŷ}_{i}-y_{i}\right)$ are the slack variables of the *i*_*th*_ observation (*i* ∈ (1, 2, …, *N*)), *y*_*i*_ is the actual wrist angle of the *i*_*th*_ observation, *N* is the number of the samples in the training set, and *C* is a constant parameter adjusting the errors. The term $\left(\xi_{i}+\xi_{i}^{*}\right)=E_{\epsilon }\left({ŷ}_{i}-y_{i}\right)$ is the ϵ-insensitive error function, where *E*_ϵ_(ŷ_*i*_−*y*_*i*_) = 0 if |ŷ_*i*_−*y*_*i*_| < ϵ. The problem was usually converted to its Lagrange dual formula for calculational simplicity. The prediction function after training was:


(12) $$\begin{array}{l} ŷ=\overset{N}{\underset{i=1}{\sum }}\left(\alpha_{i}-\alpha_{i}^{*}\right)\kappa \left(x_{i},x\right)+{ŷ}_{j}-\epsilon -\overset{N}{\underset{i=1}{\sum }}\left(\alpha_{i}-\alpha_{i}^{*}\right)\kappa \left(x_{i},x_{j}\right), \end{array}$$

where (***x***_*i*_, *y*_*j*_) is a point on the boundary of the ϵ-tube and (***x***, *ŷ*) is the input data and the prediction angle. α_*i*_ and $\alpha_{i}^{*}$ are the *i*_*th*_ Lagrangian coefficients for the two constraints. The SVR-based estimation algorithm also utilized 3 models for multiple DoFs. The estimation function was expressed as: $\hat{y_{k}}=\omega_{k}\phi_{k}\left(x\right)+b_{k}$, *k* = 1, 2, 3, where $\hat{y_{k}}$ is the estimated angle of the *k*_*th*_ DoF, **ω**_*k*_ is the *k*_*th*_ weight vector, and $\beta_{0k}^{\prime }$ is the corresponding intercept. The kernel function *ϕ*_*k*_**(*****x*****)** was also trained separately for each DoF.

### 3.4. Evaluation Method

#### 3.4.1. Cross-Validation

We used cross-validation (CV) to evaluate the estimation results. Data of one session were used for training, the fitted regression function, and the selected optimal features were tested on the data of the other session. The procedure repeated twice until all the data were used for training and testing once. The 2 results were then averaged as the final results.

#### 3.4.2. NRMSE and RMSE

The normalized root mean square errors (NRMSE) were calculated for each subject. The NRMSE was expressed as:


(13) $$\begin{array}{l} NRMSE=\frac{\sqrt{\frac{1}{N}\sum_{i}^{N}{\left(\hat{y\left(i\right)}-y\left(i\right)\right)}^{2}}}{y_{max}-y_{min}}, \end{array}$$

where $\hat{y\left(i\right)}$ is the *i*th calculated data point by the regression model, *y*(*i*) is the *i*th actual data point, and *N* is the number of points in total. *y*_*max*_ and *y*_*min*_ are the maximum and minimum values of the actual data points. The value changed between 0 and 1, with smaller values indicating better results. The numerator of Equation (13) was RMSE. RMSE represents the difference between the reference value (wrist angle) and the estimated one. The unit of the RMSE was the angle (°).

#### 3.4.3. Coefficients of Determination

The coefficients of determination (*R*^2^) was defined as:


(14) $$\begin{array}{l} R^{2}=1-\frac{\sum_{i}{\left(y_{i}-\hat{y_{i}}\right)}^{2}}{\sum_{i}{\left(y_{i}-ȳ\right)}^{2}}, \end{array}$$

where *y*_*i*_ is the *i*_*th*_ point of the reference data, and $\hat{y_{i}}$ is the corresponding estimated one by the EIT signals. *y*_*i*_ is the angle of the IMU. $ȳ=\frac{1}{N}\sum_{i}y_{i}$ is the average value of the reference angle. *N* is the number of points in total.

#### 3.4.4. Statistical Analysis

We conducted one-way repeated measure analysis of variance (ANOVA) to compare the estimation results. The independent factors were the regression models and features, respectively. The dependent factor was the estimation results (*R*^2^, NRMSE, and RMSE). The significance value was 0.05 (α = 0.05).

#### 3.4.5. Parameter Fitting

The parameters λ and *C* are selected with the parameter optimization procedures with the training data. For each subject, we used three-fold cross-validation on the training data to optimize the parameters. The metric was mean square error (MSE). The parameter with the smallest MSE would be selected as the optimal one, and used to predict the wrist angles on the testing data set.

## 4. Results

### 4.1. Overall Estimation Results

**Figure 4:**
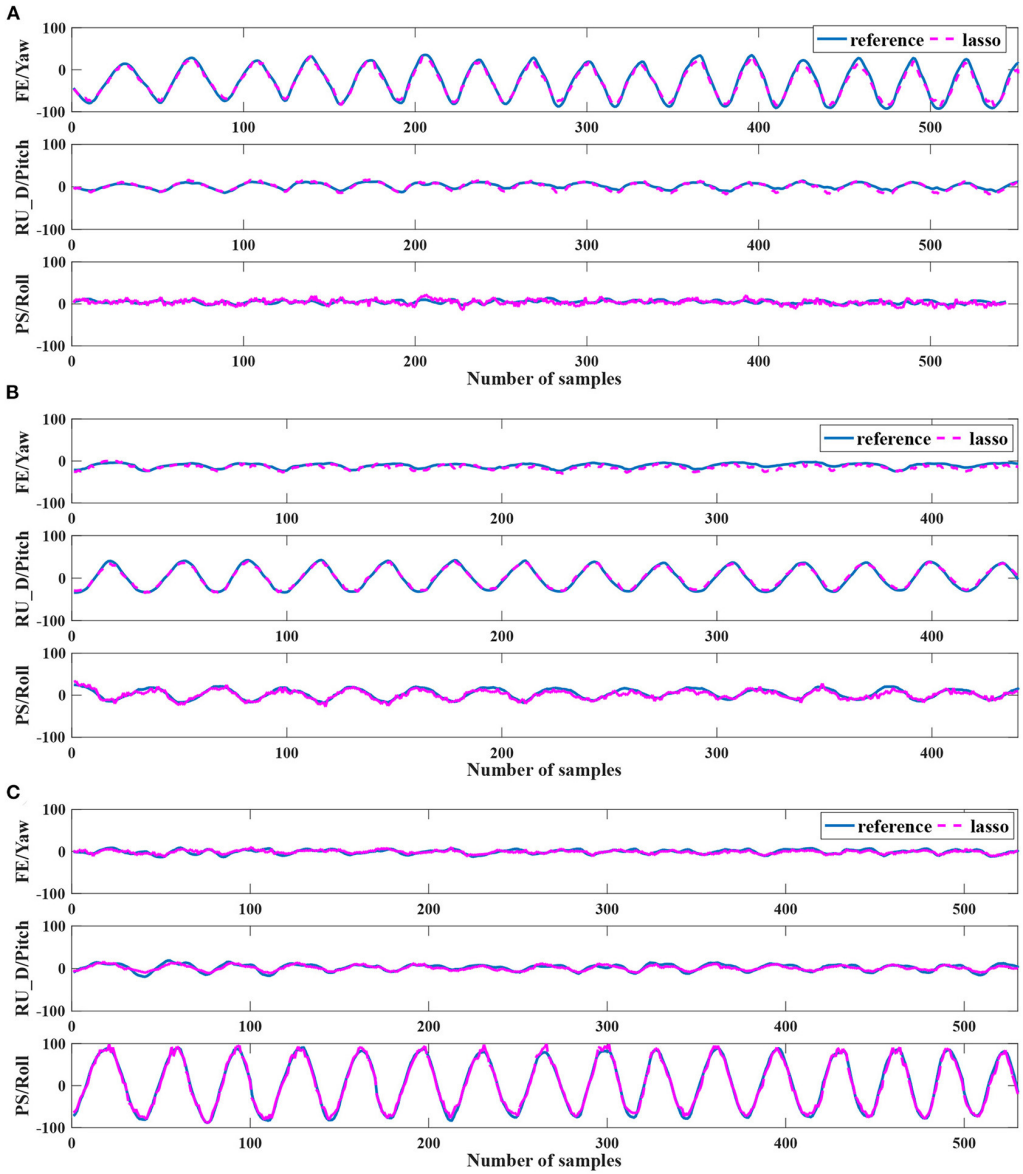
The pseudo real-time estimation results of Subject 12. **(A–C)** show the case result of Exp1, Exp2, and Exp3, respectively. The blue curves are the reference angles measured by the IMU sensors. The violet dotted curves are the estimated angles by the EIT signals. The estimated curves are calculated by the Lasso with the augmented feature.

The estimation results for each subject with different regression models (Lasso and SVR) and features (***S***, $\overset{-}{S}$) are shown in Table 1. The kernel-based SVR produced the smallest average RMSE with normal features ***S***, and the average value was 7.44 ± 1.30°. In addition to RMSE, the best average performances of NRMSE and *R*^2^ values were produced by Lasso with augmented features $\overset{-}{S}$. The smallest average NRMSE value was 0.053 ± 0.008, and the highest average *R*^2^ value was 0.92 ± 0.04. A repeated-measures ANOVA with a Greenhouse-Geisser correction indicated that the regression models did not elicit statistically significant differences in estimation results, *F*_(1, 0.16)_ = 0.81, *p* = 0.37. We also compared the results of different features (***S***, $\overset{-}{S}$) with one-way repeated measure ANOVA. For SVR, there was no evidence that the estimation results are significantly influenced by the features (*p* > 0.05). For Lasso, the average RMSE was not significantly affected by the features (*p* = 0.21). The NRMSE and *R*^2^ were significantly different with different features (*p* < 0.05). In other words, using augmented features $\overset{-}{S}$ significantly increased the estimation performances in NRMSE and *R*^2^. Among the subjects, Subject 12 was an outlier, which performed extremely badly in RMSE, but the results of NRMSE and *R*^2^ were at the same level as that of others. The large range of motion partly caused the large RMSE (see Figure 4). Excluding the results of Subject 12, Lasso with the augmented features produced the lowest RMSE among the regression models and features.

**Table 1:** Overall estimation results for each subject.

	**Lasso**						**SVR**					
	**RMSE(°)**		**NRMSE**		***R* ^2^**		**RMSE(°)**		**NRMSE**		***R* ^2^**	
**Subjects**	***S***	$\overset{-}{S}$	***S***	$\overset{-}{S}$	***S***	$\overset{-}{S}$	***S***	$\overset{-}{S}$	***S***	$\overset{-}{S}$	***S***	$\overset{-}{S}$
Subject 1	6.31	5.33	0.051	0.044	0.93	0.95	5.22	5.82	0.043	0.047	0.95	0.94
Subject 2	6.67	6.83	0.059	0.060	0.89	0.89	6.25	5.61	0.057	0.052	0.90	0.91
Subject 3	7.26	6.02	0.064	0.054	0.87	0.91	7.18	7.40	0.063	0.064	0.88	0.88
Subject 4	8.82	7.99	0.068	0.061	0.83	0.86	8.44	9.32	0.064	0.071	0.85	0.82
Subject 5	6.05	6.15	0.037	0.037	0.96	0.97	6.94	6.39	0.047	0.039	0.93	0.96
Subject 6	10.30	9.13	0.066	0.055	0.90	0.94	9.71	8.80	0.060	0.053	0.93	0.95
Subject 7	7.80	7.07	0.056	0.051	0.92	0.93	7.30	8.02	0.051	0.059	0.93	0.90
Subject 8	9.58	8.70	0.077	0.068	0.80	0.85	9.42	8.27	0.075	0.065	0.81	0.86
Subject 9	7.20	6.72	0.055	0.049	0.92	0.94	7.09	7.36	0.054	0.053	0.92	0.93
Subject 10	7.41	6.68	0.057	0.051	0.94	0.95	6.89	6.76	0.054	0.053	0.94	0.94
Subject 11	7.01	7.47	0.052	0.053	0.92	0.92	6.54	8.72	0.049	0.060	0.93	0.89
Subject 12	11.31	13.35	0.054	0.049	0.94	0.95	8.28	10.65	0.042	0.048	0.96	0.95
AVE	7.98	7.62	0.058	0.053	0.90	0.92	7.44	7.76	0.055	0.055	0.91	0.91
STD	1.66	2.11	0.010	0.008	0.05	0.04	1.30	1.50	0.010	0.009	0.04	0.04

### 4.2. Detailed Estimation Results

We analyzed the results of Lasso in more detail as it produced better estimation results overall than SVR. The results with normal features ***S*** and the augmented features $\overset{-}{S}$ are shown in Tables 2, 3, respectively. In each row, the estimation results were calculated from the model trained for the corresponding DoF, expressed as the plane's motion (*XoY*, *YoZ*, and *XoZ*). Each column was the estimation results tested with the data (F/E, RD/UD, and P/S). According to the experimental setups (left part of Figure 2), F/E occurred in the plane of *XoY*, RD/UD occurred in the plane of *YoZ*, and P/S occurred in the plane of *XoZ*. The principle DoF of the model, i.e., the model trained with the data of F/E and tested on the angle in the plane of *XoY*, produced the lowest NRMSE and highest *R*^2^ values among all the other combinations. The models with augmented features produced slightly better results than normal features. The average NRMSE of the diagonal was 0.06.

**Table 2:** Estimation results with Lasso and normal features.

	**Estimation**								
	**RMSE(°)**			**NRMSE**			***R* ^2^**		
**Training**	**F/E**	**RD/UD**	**P/S**	**F/E**	**RD/UD**	**P/S**	**F/E**	**RD/UD**	**P/S**
*XoY*	8.2 ± 4.19	4.74 ± 1.02	9.34 ± 2.7	0.06 ± 0.03	0.22 ± 0.1	0.41 ± 0.2	0.94 ± 0.06	0.27 ± 0.25	0.07 ± 0.13
*YoZ*	7.96 ± 5.16	4.08 ± 2	11.64 ± 8.16	0.34 ± 0.23	0.06 ± 0.03	0.36 ± 0.15	0.05 ± 0.16	0.95 ± 0.04	0.13 ± 0.31
*XoZ*	7.37 ± 3.33	3.95 ± 1	12.19 ± 3.66	0.36 ± 0.17	0.18 ± 0.05	0.07 ± 0.02	0.11 ± 0.26	0.34 ± 0.35	0.94 ± 0.04

**Table 3:** Estimation results with Lasso and augmented features.

	**Estimation**								
	**RMSE(°)**			**NRMSE**			***R* ^2^**		
**Training**	**F/E**	**RD/UD**	**P/S**	**F/E**	**RD/UD**	**P/S**	**F/E**	**RD/UD**	**P/S**
*XoY*	8.82 ± 5.13	3.75 ± 1.1	7.12 ± 1.59	0.06 ± 0.04	0.17 ± 0.08	0.31 ± 0.17	0.93 ± 0.08	0.45 ± 0.3	0.14 ± 0.23
*YoZ*	7.43 ± 5.43	3.71 ± 2.12	12.25 ± 14.81	0.31 ± 0.24	0.05 ± 0.03	0.31 ± 0.14	0.05 ± 0.16	0.96 ± 0.04	0.13 ± 0.32
*XoZ*	5.30 ± 2.30	3.27 ± 0.94	12.16 ± 4.04	0.26 ± 0.1	0.15 ± 0.04	0.07 ± 0.02	0.18 ± 0.29	0.47 ± 0.35	0.94 ± 0.04

The multi-DoF motion of the wrist joint was coupled in muscular contractions. Training with the data of 1-DoF could not produce equally well results for the same DoF in other motions. The non-diagonal results were worse than that of the diagonal ones. For the non-diagonal results, the augmented features produced more minor estimation errors (RMSE and NRMSE) and higher *R*^2^ values than that of using normal features. For the multi-DoF models, the models trained for *XoY* and *XoZ* both produced relatively low NRMSE values for the motion of RD/UD, which were 0.17 and 0.15 for *XoY* and *XoZ* (augmented feature), respectively. However, the models could not generalize well to the other motions.

### 4.3. Feature Importance of the Estimation Models

**Figure 5:**
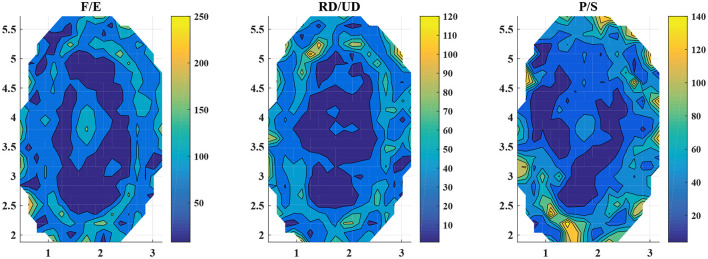
Visualization of the augmented features after training. The three subplots demonstrate the visualization of the three models, from left to the right, F/E, RD/UD, and P/S, respectively. The color bars denote the times that the elements were selected in the L1-regularization. The regions of the warm color were selected more times than that of the cold colors. The data were summarized by all the subjects.

The L1-regularization in Lasso clipped the small weights to prevent overfitting. We analyzed the trained models of Lasso with the augmented features, which produced the lowest estimation errors. The dimension of the features (number of non-zero elements in **ω**_*k*_ as described in section 3.3.1) is shown in Table 4. In the table, F/E, RD/UD, and P/S are the results of the models trained with the motion of F/E, RD/UD, and P/S, respectively. The average dimensions across the subjects were 516.9 ± 77.3, 421.9 ± 113.4, and 441.4 ±, 222.4 for the model of F/E, RD/UD, and P/S, respectively. The dimensions of the features took up 0.3% to 1.2% of the dimension of the augmented feature $\overset{-}{S}$. In Table 4, F/E_Diag, RD/UD_Diag, and P/S_Diag are the numbers of non-zero elements in the diagonal of the matrix ***S***^¬^. The average values across the subjects were 10.8 ± 4.4, 13.0 ± 5.1, and 6.7 ± 4.2 for F/E, RD/UD, and P/S, respectively. The numbers of the diagonal elements only took up <5% of the total dimensions of the regularized augmented features. The results suggested that information of the inter-regions in the EIT images was more relevant to the estimation results in multi-DoF estimation. We visualized the augmented features after L1-regularization of Lasso (see Figure 5). According to Equation (8), an element of the augmented feature $\overset{-}{S}$ can be decomposed into two elements of the normal features ***S***_*i*_ and ***S***_*j*_ (*i, j* ∈ 1, 2, 3, …, 382). The two decomposed elements can be visualized on the corresponding square regions of the EIT segmented image. We repeatedly plotted the decomposed elements (a unit color for each element) on the EIT image for all the subjects, in which the selected elements were accumulated. The times that the selected features fell on the region were indicated by the color bars (Figure 5). For all three models, the inner parts were less likely to be chosen, which corresponded to the areas of the bones. For the features of F/E, there were no obvious regions frequently selected for regression. For the other two motions, the top regions (RD/UD) and bottom-left regions (P/S) were more frequently chosen than the other parts.

**Table 4:** Dimension of features after Lasso regularization.

**Subjects**	**F/E**	**F/E_Diag**	**RD/UD**	**RD/UD_Diag**	**P/S**	**P/S_Diag**
1	442	18	272	5	288	2
2	431	7	271	7	254	3
3	458	6	536	11	621	10
4	473	6	307	8	398	7
5	451	7	382	10	849	7
6	574	10	524	20	285	2
7	590	10	506	19	878	17
8	512	12	538	19	288	7
9	637	11	312	16	297	9
10	523	16	363	14	395	3
11	464	9	537	16	288	7
12	648	18	515	11	456	6

## 5. Discussion

### 5.1. Estimation Performances

In our study, we proposed an EIT-based method for 3-DoF wrist kinematics estimation. The approach took advantage of the human forearm anatomical features measured by the EIT system, extending the measurement scope of the muscle-signal-based interfaces. In our study, the Lasso-based regression model produced an average RMSE of 7.62° with the augmented EIT features across 12 subjects (1:1 CV). The average *R*^2^ value was 0.92. The approach achieved comparable results to that of state-of-the-art. As mentioned in section 1, the estimation performances of continuous wrist kinematics are determined by many factors, including the number of DoFs, the system setups, the algorithms, and evaluation method (Muceli and Farina, 2012; Liu et al., 2017; Bakshi et al., 2018; Gao et al., 2018; Kapelner et al., 2019; Shahzad et al., 2019; Yang et al., 2019; Ameri, 2020; Bao et al., 2020, 2021; Zhao et al., 2020). The authors of the studies used high-density sEMG systems to record forearm muscle signals for simultaneous multi-DoF wrist angle estimation when the subjects performed random motions. Due to the task complexity, the estimation accuracies (represented by the *R*^2^ values) range from 0.77 to 0.90. In the study of Bakshi et al. (2018), the authors sampled 8-channel sEMG signals from the forearm and designed a kernel least square tracker based algorithm for 3-DoF wrist angle estimation. The motion tasks included the basic wrist motions the same as that of our study. With 10-fold CV, the study (Bakshi et al., 2018) produced an average *R*^2^ values ranging from 0.90 to 0.92 across 10 healthy subjects for the dominant DoFs (training and testing with the data of the same DoF). The studies of Shahzad et al. (2019) proposed a linear regression cascade decoder for 3-DoF upper-limb angle estimation (one DoF for each joint). The average RMSE of wrist joint (flexion/extension) angle estimation was 11.51° across 10 healthy subjects. In the studies of Gao et al. (2018), Yang et al. (2019), Ameri (2020), Bao et al. (2020, 2021), the authors designed machine-learning-based algorithms for wrist angle estimation. In the study of Bao et al. (2021), the authors designed a deep Kalman filter network based on 5-channel sEMG signals. With 4:1 CV, the algorithm produced an average RMSE of 14.5° across 8 healthy subjects on flexion/extension tasks. The study of Bao et al. (2020) addressed the issues of multi-DoF task using CNN-LSTM, which produced the *R*^2^ value as high as 0.89 (F/E) in multi-DoF tasks. (Ameri, 2020) proposed a polynomial-based method for 3-DoF wrist angle estimation (6 basic motions and 8 combinations of them) and produced the average *R*^2^ values ranging from 0.82 (P/S) to 0.88 (F/E) across 10 healthy subjects. In addition to machine learning, the musculoskeletal model based algorithms were also designed and evaluated on the one-DoF task (wrist flexion/extension) (Zhao et al., 2020). The average RMSEs across 8 healthy subjects ranged from 10.8° to 17.59° with different experimental setups.

In addition to sEMG-based approaches, there are some researchers conducting US-based studies on similar forearm motion estimation/recognition tasks as our study (Shi et al., 2009; Castellini, 2014; Akhlaghi et al., 2015; Yang et al., 2020a,b). The study which addressed one-DoF wrist angle estimation task (Shi et al., 2009) yielded the average *R*^2^ values ranging from 0.94 to 0.96 with varying extension rates across seven subjects. The researchers of the study (Castellini, 2014) achieved finger angle/force estimation with transverse US images of the forearm. An average error around 2% of the sensor range (data glove or force sensor) was obtained. In the study of Akhlaghi et al. (2015), the authors measured transverse forearm muscle with an US device and classified 15 different hand motions. The average classification accuracy was 91–92%. On the other side, the US device is burdensome in the probe and the signal processing system. To increase the compactness of the technique for wearable uses, some researchers developed the wearable US front-ends for human–machine interfaces, achieving accurate wrist motion estimation (Yang et al., 2020a,b). According to the study of Yang et al. (2020a), wrist tracking precision (*R*^2^) of 0.954 ± 0.012 and a finger gesture classification accuracy of 96.5 ± 1.7% can be simultaneously achieved. In the study of Yang et al. (2020b), the average *R*^2^ value for wrist pronation/supination and hand closing/opening estimation was 0.975–0.983 with ipsilateral training.

The improvement of our study over the existing muscle-signal-based interfaces (sEMG and US) is that we provided an alternative solution to wrist kinematics estimation unobtrusively. The proposed EIT-based interface extent the measurement scope of sEMG sensors for deep muscle information and reduced the obtrusiveness of the US probe by the soft-elastic sensing band. Compared with our previous study (Zheng et al., 2021) which estimated one-DoF wrist angle with the musculoskeletal model, our current study extends the EIT-based interface to multi-DoF tasks, increasing the task complexity.

### 5.2. Confounding Factors

#### 5.2.1. Training Data

The training data distribution strongly influenced the estimation results. The forearm muscle contractions are coupled in multi-DoF motions. As shown in Tables 2, 3, the results on the diagonal are much better than that of the off-diagonal for both normal features and augmented features. The training data on the diagonal were sampled with the full-range motion of that DoF, such that the regression model was intensively trained. The off-diagonal results of RD/UD were better than that of the other two DoFs (RMSE and NRSME). One explanation was that the range of motion was much smaller than F/E and P/S. With the proposed model, training with full-range F/E and P/S is necessary to get minor estimation errors.

#### 5.2.2. Augmented Features

Using augmented features $\overset{-}{S}$ significantly reduced the estimation errors (as shown in Table 1) than using the normal features. In the detailed estimation results (Tables 2, 3), augmented features mainly act in the off-diagonal results, i.e., training/testing with different DoF motion data. The biological relevance was that the EIT features (of different regions) correspond to the anatomical muscle contractions by constructing the conductivity changes. Multiple muscle coordinations controlled the wrist joint motion. The augmented features extracted the muscle coordinations by multiplication of different EIT regions.

#### 5.2.3. Regression Model

The linear model outperformed the non-linear one with the augmented feature in our wrist angle estimation task. One possible reason was that the Lasso used L1-regularization to optimize the model, which totally removed an unnecessary term of the model rather than assigned it with a tiny weight as the SVR did. The regression model of Lasso was less likely to be over fitted than that of SVR when calculated on the testing data set. Another possible reason was the biological significance of the EIT features. It is possible that the muscle coordination (conveyed by the multiplication of different EIT regions) is linearly related to the wrist motions. In future study, the biological significance of the sensing approach will be extensively investigated by comparisons with the reference signals (such as MRI).

### 5.3. Limitations and Future Works

The study is limited in the following aspects. First, the subjects performed relatively structured wrist motion in our study, including F/E, P/S, and RD/UD. The random simultaneous motion estimation tasks have yet to be addressed. The muscle contractions are coupled in controlling the wrist joint motions. One muscle can act in multiple DoF motion control. The arm postures can also influence the muscle signals due to the gravity changes. The generalization ability of the regression model should be increased to meet the task complexity. Second, in our study, the estimation method was designed based on machine learning algorithms. The estimation results were affected by the training data distribution. Although the augmented feature took advantage of the spatial information of EIT signals, the biological significance within the EIT features remained to be systematically investigated. In future works, the following issues will be addressed. First, the relationships between the EIT features and the anatomical muscle parameters will be quantitatively investigated. The anatomical muscle distribution will be implemented in the estimation models as the prior information. The regression models will be designed by fusing the musculoskeletal mechanism to increase the generalization ability. Second, the robustness of the EIT-based approach will be improved. The impacts of different voluntary muscle contractions, re-donning the sensing front-ends, and long-term uses on the estimation performances will be studied and alleviated. Third, the generalization ability of the regression model will be improved. The method's performances in our current study were dependent on training data distribution. The model incorporating the biological features will be designed and evaluated on simultaneous multi-DoF tasks.

## 6. Conclusion

In our study, we proposed an EIT-based approach for wrist angle estimation. The method took advantage of the muscular spatial information with augmented features and L1-norm regularization. The following conclusions can be made. First of all, the estimation results proved the feasibility of using the EIT interface for multi-DoF wrist angle estimation. The average estimation results are at the same level as that of state-of-the-art. Second, the correlation between different EIT regions conveys relevant wrist motion information. A linear kernel function (augmented feature) can significantly reduce the estimation errors in the tasks. Third, the normal features worked well for single-DoF estimation (training/testing within the same DoF). The augmented features mainly act in multi-DoF estimation tasks (training/testing in different DoFs). The main limitations of the study lie in the task complexity and model's biological significance. Future works will be focused on increasing the generalization ability of the regression model.

## Data Availability Statement

The raw data supporting the conclusions of this article will be made available by the authors. Further inquiries can be directed to the corresponding author.

## Ethics Statement

The studies involving human participants were reviewed and approved by Ethical Review Board of Institute of Automation, Chinese Academy of Sciences. The patients/participants provided their written informed consent to participate in this study.

## Author Contributions

EZ designed the research, conducted the experiments, and wrote the paper. JZ analyzed the data. JZ, QW, and HQ performed the research. All authors contributed to the article and approved the submitted version.

## Funding

This work was supported by the National Natural Science Foundation of China (No. 62073318).

## Conflict of Interest

The authors declare that the research was conducted in the absence of any commercial or financial relationships that could be construed as a potential conflict of interest.

## Publisher's Note

All claims expressed in this article are solely those of the authors and do not necessarily represent those of their affiliated organizations, or those of the publisher, the editors and the reviewers. Any product that may be evaluated in this article, or claim that may be made by its manufacturer, is not guaranteed or endorsed by the publisher.

## Footnotes

^1^EIDORS (2020). Available online at: http://eidors3d.sourceforge.net/.
